# Correlates of low birth weight in term pregnancies: a retrospective study from Iran

**DOI:** 10.1186/1471-2393-8-12

**Published:** 2008-04-19

**Authors:** Mariam Vahdaninia, Sedigheh Sadat Tavafian, Ali Montazeri

**Affiliations:** 1 Iranian Institute for Health Sciences Research, Tehran, Iran

## Abstract

**Background:**

Low birth weight (LBW) is considered as a major multifaceted public health concern. Seventy-two percent of LBW infants are born in Asia. An estimation of 8% LBW infants has been reported for Eastern Mediterranean region including Iran. This study investigated contributory factors of LBW in singleton term births in Tehran, Iran. Tehran is a multicultural metropolitan area and a sample from the general population in Tehran could be regarded as a representative sample of urban population in Iran.

**Methods:**

This was a retrospective study using data from 15 university maternity hospitals in Tehran, Iran. Data on all singleton term births in these hospitals were extracted from case records during a one calendar year. Study variables included: maternal age, maternal educational level, history of LBW deliveries, history of preterm labor, cigarette smoking during pregnancy, number of parities, chronic diseases and residential area (Tehran versus suburbs of Tehran). In order to examine the relationship between LBW and demographic and reproductive variables the adjusted logistic regression analysis was performed.

**Results:**

In all, data for 3734 term pregnancies were extracted. The mean age of women was 25.7 (SD = 5.3) years and 5.2% of term births were LBW. In addition to association between LBW and maternal age, significant risk factors for LBW were: history of LBW deliveries [adjusted odds ratio (OR) = 2.53, 95% confidence interval (CI) = 1.06–6.03], smoking during pregnancy (OR = 4.64, 95% CI = 1.97–10.95) and chronic diseases (OR for hypertension = 3.70, 95% CI = 2.25–6.06, OR for others = 2.04, 95% CI = 1.09–3.83).

**Conclusion:**

The findings indicate that in addition to maternal age, history of LBW deliveries; smoking during pregnancy and chronic diseases are significant determinants of LBW in this population. This is consistent with national and international findings indicating that maternal variables and risk behaviors during pregnancy play important roles on LBW.

## Background

Low birth weight (LBW) is a reliable indicator in monitoring and evaluating the success of maternal and child health programs and has been defined as a birth weight less than 2500 gr [1]. It is estimated that worldwide 15.5% of all live births per year are LBW and more than 95 percent of LBW infants are born in developing countries. Seventy-two percent of LBW infants are born in Asia, although large differences exist in WHO Asian regions and its sub-regions. It is estimated that there are 8% of LBW infants in Eastern Mediterranean region including Iran [2].

A baby's low weight at birth is either the result of preterm birth or of intrauterine growth retardation (IUGR) [2]. The latter implies that fetus's growth has been inhibited and thus the fetus has not attained the potential growth. The diagnosis of IUGR is usually based on small size for gestational age (SGA). However, IUGR is not completely equal to SGA and there is need to develop standardized and population-specific growth charts to well differentiate these clinical terms in practice [3].

The evidence suggests preterm birth as a main cause of newborn mortality and morbidity [4]. Indeed, LBW due to IUGR as a poor birth outcome affects the person throughout life course and is associated with a higher risk of developmental impairments including cognitive development [5], medical and health outcomes in adulthood [6,7]. Giving birth to a LBW infant is influenced by several determinants including maternal variables [8-10], socio-economic status [11] and environmental factors [12].

This study investigated the contribution of socio-demographic and reproductive-obstetrical risk factors on LBW in all singleton term births that referred from Tehran and its suburbs to the main general and teaching hospitals in Tehran during a one calendar year (2005).

## Methods

A retrospective study was conducted in 15 maternity wards of the main general and teaching hospitals in Tehran, Iran. The ethics committee of Tehran University of Medical Sciences approved the study. Using hospital records, data on all singleton term births (≥37 gestational weeks) of married pregnant women in these hospitals during year 2005 were extracted. Studied variables included maternal age, maternal education, number of parities, history of LBW deliveries (HLBW), history of preterm labor (HPL), cigarette smoking during pregnancy, chronic diseases and residential area (Tehran versus suburbs of Tehran). Mothers' chronic diseases were considered as categorical variable: 1) none (no report of any disease in case notes), 2) hypertension (most reported condition) and 3) others. The latter included renal, heart, respiratory, arthritis and several other chronic diseases. There were no other LBW related data available. Birth outcome was defined as LBW and covered IUGR as well. All term LBW babies had been examined by a gynecologist. According to sonography results and weight chart for gestational ages, the diagnosis of IUGR was established.

The regression analysis was performed to calculate crude and adjusted odds ratios and to examine the predictive effect of variables studied on risk for LBW. LBW was considered as dependent variable and was categorized into two groups: <2500 gr. and ≥2500 gr. Other variables were entered into the model as key independent variables and except "maternal age and number of deliveries"; all other variables were entered into the model as categorical data.

## Results

In all, 3734 pregnant women were studied. The mean age of mothers and the mean duration of pregnancy were 25.7 (SD = 5.3) years and 39.03 (SD = 1.36) weeks respectively. Overall, 5.2% of term births were LBW and of these 4% had been recognized as IUGR babies. The mean birth weight was 3.2 (SD = 0.47) Kg. Self-reported cigarette smoking was 1.2% (n = 45). Table 1 presents the distribution of LBW determinants (total and LBW statistics).

**Table 1:** The distribution of LBW determinants (total and LBW statistics)

	**Total**	**LBW cases**
	
	**Number (%)**	**Number (%)**
**Maternal age (at child birth)**	**(n = 3720)**	**(n = 182)**
≤20	631 (17.0)	39 (21.4)
21–34	2817 (75.7)	129 (70.9)
≥35	272 (7.3)	14 (7.7)
Mean (SD)	25.7 (5.3)	24.8 (5.6)
**Maternal education**	**(n = 3726)**	**(n = 181)**
Illiterate & primary	1350 (36.2)	64 (35.4)
Secondary & high school	2215 (59.5)	115 (63.5)
University	161 (4.3)	2 (1.1)
**Number of parities**	**(n = 3734)**	**(n = 175)**
none	4 (0.1)	1 (0.6)
1	1666 (45.7)	85 (48.6)
2	1127 (31.0)	49 (28.0)
≥3	844 (23.2)	40 (22.8)
Mean (SD)	1.91 (SD = 1.15)	1.90 (1.21)
**History of LBW deliveries**	**(n = 3731)**	**(n = 182)**
No	3578 (95.9)	169 (92.9)
Yes	153 (4.1)	13 (7.1)
**History of pre term labor**	**(n = 3722)**	**(n = 182)**
No	3582 (96.2)	173 (95.1)
Yes	140 (3.8)	9 (4.9)
**Cigarette smoking**	**(n = 3731)**	**(n = 181)**
No	3686 (98.8)	174 (96.1)
Yes	45 (1.2)	7 (3.9)
**Chronic diseases**	**(n = 3733)**	**(n = 182)**
None	3380 (90.5)	148 (81.3)
Hypertension	188 (5.0)	22 (12.1)
Others	165 (4.5)	12 (6.6)
**Residence**	**(n = 3716)**	**(n = 181)**
Tehran	2504 (67.4)	109 (60.2)
Suburbs of Tehran	1212 (32.6)	72 (39.8)

The findings of regression analysis for crude and adjusted odds ratios (OR) of LBW and its determinants are shown in table 2. It was found that significant risk factors for LBW were: maternal age (OR = 0.96, 95% CI = 0.92–1.00), positive history of LBW deliveries (OR = 2.53, 95% CI = 1.06–6.03), smoking during pregnancy (OR = 4.64, 95% CI = 1.97–10.95) and mothers' history of chronic diseases (OR for hypertension = 3.70, 95% CI = 2.25–6.06, OR for others = 2.04, 95% CI = 1.09–3.83).

**Table 2:** Logistic regression analysis for LBW and its determinants

	**Crude ORs**	Adjusted ORs *
	
	**OR (95% CI)**	**OR (95% CI)**
**Maternal age (at child birth)**	0.96 (0.94–0.99)	0.96 (0.92–1.00)
**Maternal education**		
University	1.0 (ref.)	1.0 (ref.)
Illiterate and primary	3.87 (0.93–16.00)	3.32 (0.79–13.94)
Secondary and high school	4.29 (1.05–17.54)	3.44 (0.83–14.25)
**Number of parities**	0.98 (0.86–1.12)	1.01 (0.85–1.20)
**History of LBW deliveries**		
No	1.0 (ref.)	1.0 (ref.)
Yes	1.94 (1.07–3.51)	2.53 (1.06–6.03)
**History of pre term labor**		
No	1.0 (ref.)	1.0 (ref.)
Yes	1.41 (0.70–2.84)	0.65 (0.23–1.81)
**Cigarette smoking**		
No	1.0 (ref.)	1.0 (ref.)
Yes	3.74 (1.63–8.54)	4.64 (1.97–10.95)
**Chronic diseases**		
None	1.0 (ref.)	1.0 (ref.)
Hypertension	3.11 (1.93–5.02)	3.70 (2.25–6.06)
Others	1.91 (1.03–3.53)	2.04 (1.09–3.83)
**Residence**		
Tehran	1.0 (ref.)	1.0 (ref.)
Suburbs of Tehran	1.41 (1.04–1.92)	1.24 (0.90–1.71)

* Adjusted for all variables studied in one model.

Maternal education did not show a significant relation with LBW, but increased risk was observed for less educated mothers (OR = 3.22) and those with secondary educational level (OR = 3.44). Although residential area (Tehran versus suburbs of Tehran) was not significant, an increased risk (OR = 1.24) was observed for women referred from suburbs of Tehran.

## Discussion

This paper evaluated the LBW contributions in a representative sample of singleton term births in the university hospitals in Tehran, Iran during 2005. The findings showed that most of the term LBW babies are recognized as IUGR (4% of 5.2%). Evidence suggests in developing countries most of LBW infants are due to IUGR [13]. In a simple definition, IUGR babies are considered those who are gestationally full-term (≥37 weeks) but of a birth weight <2500 gr. When there are no birth-weight-for-gestation percentiles for a population similar to the one being studied, this definition is quite useful and applicable [14].

Based on adjusted logistic regression analysis, maternal age was a protective factor for LBW and one year age increase, showed a 4% risk reduction (Table 2). A study from Zahedan, Iran on prevalence and risk factors of LBW in 1109 hospital births showed maternal younger age is related to a LBW baby [8]. Similar findings from international studies have shown lower maternal age for giving birth to a LBW infant [14]. This study provided data on a representative sample of term births in Tehran, Iran and thus would be a proper base of age effect on LBW and IUGR.

The present study indicated that obstetric history of previous LBW deliveries was a significant risk factor for LBW (OR = 2.53). The findings from this study with a relatively large sample of singleton term births in Tehran, Iran compares well to the findings from other studies. A large study by Kramer indicated risk of delivering an IUGR infant was 2.75 times greater for women with one or more previous LBW infants than for women with no history of LBW deliveries [15]. Another study from Egypt has confirmed the importance of previous history of LBW infants (after adjusting for other risk variables) on increased chance of giving birth to a LBW infant [16].

We did not find a significant relationship between LBW and number of parities (p = 0.84). Studies on the topic have shown primiparous women have a greater risk of IUGR than multiparous women [15].

Also, cigarette smoking was found as a risk factor for LBW (OR = 4.64), although it was self-reported. Evidence suggests the strong effect of cigarette smoking during pregnancy on LBW even after controlling for other variables [17-19]. Policies focused on smoking during pregnancy should be properly emphasized in maternal and child health care services by establishing interventional and educational programs.

Maternal history of chronic diseases including hypertension and other chronic conditions increased the risk of giving birth to a LBW infant by 3.70 and 2.04 folds respectively. Documented research has confirmed that maternal diseases increase the risk of delivering LBW infants [20,21].

Although, maternal educational level in illiterate-primary and secondary-high school grades increased the risk of LBW (OR = 3.32 & 3.44 respectively), there was not a significant association. Well established studies have indicated that mothers with lower educational level give birth more to LBW neonates [8,11,22].

Finally, residential area (Tehran versus suburbs of Tehran) in adjusted analysis did not show a statistical significant relationship with LBW, but an increased risk (OR = 1.24) was observed for pregnant women from suburbs of Tehran. Investigating quality of primary care may explain the region differences in LBW. Studies on LBW have suggested significant associations with socioeconomic indicators and area deprivation. Women with lower socio-economic status and those living in deprived areas give birth more to LBW infants [8,23,24].

## Conclusion

This study provided data on several risk factors for LBW and IUGR in Tehran, Iran. As LBW would induce complications during infancy period and life course, prevention and control of its determinant factors should be considered in primary health care settings in order to improve mother and child health.

## Abbreviations

LBW: Low Birth Weight; IUGR: Intra Uterine Growth Retardation; SGA: Small for Gestational Age; HLBWD: History of LBW Deliveries; HPD: History of Preterm Labor; OR: Odds Ratio; CI: Confidence Interval.

## Competing interests

The author(s) declare that they have no competing interests.

## Authors' contributions

MV and ST designed and conducted the study. MV and AM analyzed the data and wrote the paper. All authors read and approved the manuscript.

## Pre-publication history

The pre-publication history for this paper can be accessed here:


